# Incisional Hernia in Cytoreductive Surgery for Advanced-Stage Ovarian Cancer: A Single-Center Retrospective Study

**DOI:** 10.3390/cancers17030418

**Published:** 2025-01-27

**Authors:** Marta Míguez Medina, Ana Luzarraga, Sara Catalán, Úrsula Acosta, Alina Hernández-Fleury, Vicente Bebia, Sonia Monreal-Clua, Martina Aida Angeles, Giulio Bonaldo, Antonio Gil-Moreno, Asunción Pérez-Benavente, Jose Luis Sánchez-Iglesias

**Affiliations:** 1 Gynecologic Oncology Unit, Department of Gynecology, Hospital Universitari Vall d’Hebron, 08035 Barcelona, Spain; 2 Gynecologic Oncology Division, Vall d’Hebron Barcelona Hospital Campus, Universitat Autònoma de Barcelona, 08035 Barcelona, Spain

**Keywords:** ovarian cancer, incisional hernia, small bites technique, ERAS

## Abstract

Background/Objectives: An incisional hernia (IH) is a frequent postoperative complication after cytoreductive laparotomic surgery for advanced ovarian cancer (AOC). It occurs in 2–22% of patients in the first two years of follow-up, depending on the series. Although different risk factors have been described for various types of malignancies and surgeries, few studies have analyzed the risk factors for hernia development in ovarian cancer (OC). However, none have examined the role of enhanced recovery after surgery (ERAS) programs. Methods: We performed a retrospective study that included patients with AOC and primary or interval debulking surgery through a median laparotomic approach. This study was conducted in Vall d’Hebron Hospital, Barcelona, Spain, between January 2015 and December 2022. Univariate and multivariate regression analyses were conducted. Results: Of the 156 patients included, 30 (19.2%) presented with an IH. The patients with IHs were smokers in a higher proportion to non-smokers (53.9% vs. 16.1%, *p* = 0.003) and more frequently presented with wound dehiscence (34.4% vs. 15.0%, *p* = 0.026). Patients in whom negative pressure wound therapy was applied had a hernia less frequently than those who had not had it (12.5% vs. 26.7%, *p* = 0.043). Similarly, the incidence of hernia decreased when patients went through an ERAS protocol (10.1% vs. 28.8%, *p* = 0.008). In the multivariate analysis, smoking was the only independent risk factor (RR 10.84, CI 2.76–42.64), and applying an ERAS protocol was seen to be the sole protective factor (RR 0.22, CI 0.08–0.61) against the development of an IH. Conclusions: The implementation of ERAS is highly recommended due to its numerous benefits, most notably the reduction in hernia incidence. Additionally, the preoperative identification of current smokers provides an opportunity for smoking cessation and targeted respiratory prehabilitation, both of which further contribute to IH reduction.

## Simple Summary

The risk of hernia in patients that undergo cytoreductive laparotomic surgery for advanced ovarian cancer is high, ranging from 2 to 22% of cases. This topic is of great interest and has been very little studied. This is especially so with regard to the surgical closure method and the analysis of the specific risk factors for this group of patients. The aim of our study was to analyze the incidence of hernia in advanced ovarian cancer and identify the associated risk factors. Another aim was to evaluate the effectiveness of ERAS programs in reducing the development of hernias, due to the great impact this complication has on our patients.

## 1. Introduction

Ovarian cancer (OC) treatment is based on the combination of cytoreductive surgery and the administration of chemotherapy with carboplatin and paclitaxel [1]. Cytoreductive surgery can be performed upfront (primary debulking surgery, PDS), or after neoadjuvant chemotherapy (interval debulking surgery, IDS), with similar prognostic outcomes [2,3,4]. In both cases, the goal is the achievement of a complete cytoreduction, as the absence of residual tumor is a key prognostic factor [5,6,7,8,9,10,11].

The surgical approach in cytoreductive surgery is a xipho-pubic laparotomy. The laparotomic approach has been associated with the development of incisional hernias in various malignancies and surgical procedures [12,13].

However, the reported rates of hernias in laparotomic surgery for ovarian cancer are highly variable, ranging from 2% to 22% depending on the series. Nearly half of those hernias develop within the first two years after surgery [13].

Several factors have been related to the appearance of incisional hernias, especially in midline incisions, such as obesity, advanced age, smoking, diabetes, the type of abdominal wall closure technique, and surgical site infections [12,14]. Additionally, patients with ovarian cancer often present with malnutrition, a proinflammatory tumoral state, and the use of adjuvant chemotherapy, all of which could even further increase the risk of hernia [15,16,17]. However, very few studies have specifically analyzed the risk factors for hernias in OC [18,19].

Enhanced recovery after surgery (ERAS) programs have been recommended for gynecological cancer surgeries, as they have been shown to decrease postoperative complications and reduce the hospital length of stay [20,21]. No evidence is currently available regarding the impact of ERAS on reducing the rate of hernias.

Given the variability in hernia rates, the potential risk factors, and the lack of specific evidence on the impact of ERAS, the aim of this study was to analyze the incidence of hernias in OC and identify the associated risk factors. Another aim was to evaluate the effectiveness of ERAS programs in reducing the development of hernias.

## 2. Materials and Methods

This is a retrospective single-center observational study including consecutive women that underwent cytoreductive surgery by the midline laparotomic approach for a preoperative suspicion of advanced ovarian cancer (either PDS or IDS) between January 2015 and December 2022 in the specialized Oncological Gynecology Unit of the Vall d’Hebron Hospital in Barcelona, Spain. Patients that underwent surgery for relapsed ovarian cancer (secondary or tertiary cytoreductive surgeries), with a history of abdominal hernia, with no complete cytoreduction, an absence of CT scans in the follow-up, or with incomplete medical reports were excluded from the study.

### 2.1. Preoperative Work-Up and Surgical Technique

Patients with suspected OC underwent a gynecologic ultrasonographic evaluation by an expert ultrasonographer, as well as a thoracic–abdominopelvic computed tomography (CT) scan and an exploratory laparoscopy for biopsy collection before the cytoreductive surgery. All the patients that underwent cytoreductive surgery since 2018 have followed an ERAS program, which includes preoperative nutritional optimization, the recommendation to stop toxic consumption, fasting only 6 h before surgery with maltodextrin administration 3 h before it, the avoidance of long-acting sedatives, and a rectal enema if intestinal resection was expected. Intraoperatively, the standard combined anesthetic, active prevention of hypothermia during the procedure, goal-directed fluid therapy based on continuous cardiac patient monitoring, avoidance of routine nasogastric intubation and abdominal drainages, and dexamethasone in induction and ondansetron after the operation for postoperative nausea and vomiting were administered. Postoperatively, an early feeding protocol was instituted, along with full mobilization in the first 72 h, non-opioid analgesia administration, and early urinary catheter removal [22]. Those patients were included in the PROFAST clinical trial published by our group [21]. Patients included from 2015 to 2018 did not go through an ERAS program.

Cytoreductive surgery was conducted via midline laparotomy in all patients. The technique of abdominal wall closure was not standardized during the study period. The type of suture (continuous or interrupted) and material (monofilament or multifilament, absorbable or no resorbable, and the size of suture) varied depending on the surgeon and the inclusion period. Negative pressure wound therapy in the skin incision was systematically applied in all patients from 2018 onward.

### 2.2. Patient’s Follow-Up

Patient follow-up was based on clinical evaluation, blood tests with tumor markers, and imaging techniques (CT-scan) every 4 months the first year, every 6 months the second year, and yearly from the third year. All CT scans (from the first post-operative CT to the last follow-up CT) were retrospectively reviewed by an expert radiologist. The CT scan was selected as the preferred imaging technique due to its high accuracy in hernia evaluation (a PPV ranging between 85% and 95% and PNV ranging between 90% and 98%) [10].

Radiologically, the diagnosis of a hernia was defined by the presence of a protrusion of soft tissue (with fat or intestines) through the fascia on the abdominal wall. In those patients, the presence of symptoms associated with a hernia was retrieved from medical records.

Data were collected from electronic patient charts, and anonymized information was input into an electronic database. Information on the baseline and demographic characteristics, including age, comorbidities, previous abdominal surgeries, presenting symptoms, and analytical values, were retrieved. Smokers were defined as individuals who have smoked daily during the past month, regardless of the quantity of cigarettes. Non-smokers include ex-smokers (those who have not smoked in the past 12 months) and individuals who have never smoked. Surgical factors, including type of surgery, blood loss, duration of surgery, intra- and post-operative complications, and length of hospital stay, were also recorded. The surgical technique, as well as the type and size of the suture utilized for abdominal closure, were also listed. Post-operative factors like the need for transfusion, the use of noradrenaline support, and intensive care unit (ICU) admission were also registered.

### 2.3. Statistical Analysis

We employed standard statistical methods to characterize the sample, including measures like the mean, standard deviations, median, interquartile range, and frequencies, depending on the distribution of the variable. Bivariate analyses were conducted to compare the distributions between the two arms using non-parametric methods. Among the factors exhibiting statistically significant differences, a correlation analysis using the Spearman correlation coefficient was performed. Factors associated with the arms that were not highly correlated with each other were retained. Based on this criterion, five potential risk factors (body mass index, ERAS, smoking status, ascites, and wound dehiscence) were identified. To explore the multivariable associations, a backward stepwise regression was conducted. A logistic regression model was put in place to estimate the risk ratio, initially including all covariates. Subsequently, covariates were systematically removed, one by one, until the final model was arrived at, guided by their significance levels (*p*-value < 0.05). All statistical analyses were conducted using R version 4.3 or above, with a significance level set at 0.050.

## 3. Results

### 3.1. Basal Characteristics

In total, 240 patients underwent a laparotomic surgery for suspected advanced ovarian cancer during the study period. However, 84 patients were excluded from the study for the following reasons: previous hernias (N = 10), residual disease after surgery (N = 41 patients), absence of CT scans at the specified time points for hernia diagnosis (N = 22), and incomplete medical records (N = 11).

Of the 156 patients included, 30 (19.2%) presented with an incisional hernia diagnosed by CT scan. The mean age at surgery was 59 years old, and the median body mass index (BMI) was 24.20 [IQR 21.95, 27.30]. Of those 156 patients, 91 (58.3%) underwent PDS and 65 (41.7%) IDS. We found no differences between the hernia and no-hernia group in terms of the basal characteristics, except for a higher incidence of smoking in the patients who developed a hernia (23.3% vs. 4.8% respectively, *p* = 0.003). The presence of a previous median laparotomy was more frequent among patients with an incisional hernia (16.7% vs. 13.5%), while patients that did not develop a post-operative hernia more often had a history of Pfannenstiel incisions (transverse incision over the symphysis pubis). The most frequently registered FIGO stage was IIIC (n = 63, 40.4%). High-grade serous ovarian cancer (HGSOC) was the most common histology (N = 120, (76.9%)). See Table 1.

### 3.2. Surgical Technique

Surgical complexity, operative time, and intraoperative blood loss were similar between groups. Regarding the technique of abdominal wall closure, the type of suture and suturing technique were similar for both groups, even though we observed a trend towards a higher use of non-resorbable sutures in the hernia group (34.8% versus 15.0%, *p* = 0.058).

In both groups, drainage (subcutaneous or abdominal) was left the same. Notably, patients who did not develop hernias had negative pressure wound therapy more frequently when compared to the other group (56.0% vs. 33.3%, *p* = 0.043). See Table 2.

### 3.3. Postoperative Course

As for the postoperative course and complications, patients with incisional hernias showed a higher rate of peritonitis and wound infection when compared to the other group (37.5% vs. 26.1% and 50.0% vs. 13.0%). They also had a significantly higher rate of wound dehiscence (37.9% vs. 17.1%, *p* = 0.026). The hernia group also had more ICU admissions (10.0% vs. 4.8%, *p* = 0.510), as well as noradrenaline use, during postoperative recovery (30.0% vs. 21.6%, *p* = 0.461).

Finally, hernias showed an incidence of 11.4% in the ERAS group vs. an incidence of 28.8% in the non-ERAS group (*p* = 0.004). Presented differently, ERAS was applied in 60.2% of cases that did not develop a hernia compared to 29.6% in the group that developed one (*p* = 0.008). See Table 3.

The mean time to an incisional hernia was 33.4 months. Most of these (76.7%) were umbilical hernias. These were followed by epigastric hernias (16.7%) and, finally, infraumbilical location (6.7%). The mean size of hernias was 62.3 mm. Among patients who developed a hernia, only three (10.0%) presented with associated symptoms and required surgery for hernia repair with mesh collocation. Case #1 presented an umbilical hernia of 136 mm; case #2 presented an umbilical hernia of 40 mm; and finally, case #3 presented an umbilical hernia of 170 mm.

### 3.4. Multivariate Analysis

**Figure 1:**
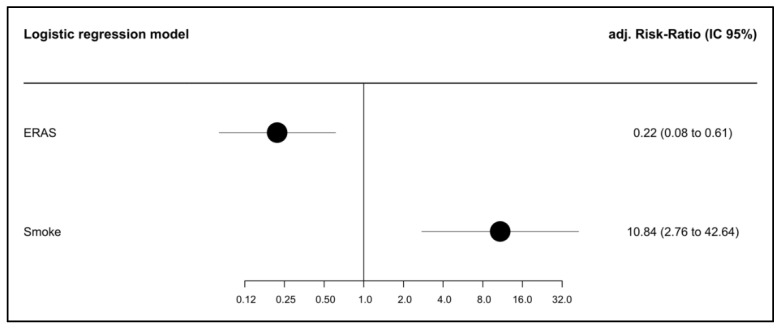
Forest plot of the multivariable model.

In the univariate analysis, only the BMI, smoking, ascites, ERAS, and surgical wound dehiscence showed an association with incisional hernias. In the multivariable model, smoking was the only independent predictor for a hernia (RR of 10.84, CI 2.76 to 42.64). Conversely, the ERAS protocol emerged as the sole protective factor against the development of a hernia (RR of 0.22, CI 0.08 to 0.61). See Figure 1.

## 4. Discussion

Our study found that 19% of patients diagnosed with advanced ovarian cancer that underwent cytoreductive laparotomic surgery would have an incisional hernia in the three years following the surgery, and 10% of them would require surgical repair due to symptoms. The multivariate analysis showed that smoking is a risk factor for the development of an incisional hernia. In contrast, the application of an ERAS protocol emerges as a protective factor.

The incidence of incisional hernias in this study is consistent with that of previous studies reporting on patients with AOC and laparotomic debulking surgery. Spencer et al. described a 10% incisional hernia rate within the first year after surgery and an additional 7.9% during the second year of follow-up [16]. Similarly, Long et al. described a 1-year hernia rate of 8.8%, rising up to 23.4% after two years of follow-up [18]. In the case of Celiksoy et al., a higher incidence of hernias was found (26.7%). This was probably due to the inclusion of primary and recurrence surgeries [19]. The main reason for excluding patients with secondary cytoreductive surgeries from our study was to avoid an overestimation of the rate of hernias. In our study, 10% of the patients with a hernia were symptomatic. However, we believe that this rate is highly underestimated, as symptoms related to a hernia could be reported or not in the clinical history. Thus, these data should be considered carefully.

Traditional risk factors reported in the literature for an incisional hernia include an advanced age, high BMI, chronic pulmonary disease, smoking, and wound infection. Elderly patients, especially those above 65 years old, have shown an increased incidence of incisional hernias, probably related to factors affecting fascial tensile strength [23,24,25]. Obesity increases intra-abdominal pressure, which can weaken the abdominal wall, and excess fat can interfere with proper wound healing, which makes a high BMI a risk factor for an incisional hernia [23,24,25]. In this study, the mean age at surgery was 59 years old, far below 65 years old. Similarly, the median BMI was 24 in the cohort, with no differences between the groups. It is also distinct from the median BMI of previous studies reporting obesity as a risk factor for a hernia [18,20]. Therefore, we could not demonstrate an association between age and weight and the development of hernias. In line with previous reports, our cohort exhibited a higher percentage of wound infection in the group that developed a hernia when compared to the ones who did not. Another risk factor that appeared statistically significant for hernias in our univariate analysis was wound dehiscence. Logically, wound dehiscence can lead to weakened tissue integrity at the incision site. This compromised area is more prone to hernias as it lacks the necessary strength to contain the abdominal contents [26].

When considering articles exclusively including cases of cytoreductive surgery for ovarian cancer, poor nutritional status has also been associated with the appearance of an incisional hernia [16]. The physical and nutritional health of oncological patients is compromised, and this seems to negatively affect the process of tissue repair. Hypoalbuminemia, mostly defined as preoperative albumin <3.5 g/dL, has been proved as an independent predictor of poor perioperative outcomes in women who have undergone open surgery for gynecologic malignancies [27]. Since 2018, a preoperative optimization of physical and nutritional health is performed as a part of the ERAS program. Consequently, in this study, levels of albumin in patients suitable for surgery were over the recommended (4.10 (IQR 3.80–4.30). Only six patients (0.04%) reached the surgery with hypoalbuminemia, and this minimal representation might explain why hypoalbuminemia was not related to a hernia in our study.

Modifiable intraoperative factors such as the technique of abdominal wall closure have been analyzed [14,20,28,29]. A randomized trial comparing smaller bites of 5 mm every 5 mm of the aponeurosis without the incorporation of fat or muscle using a 2–0 polydioxanone suture (slowly absorbable suture) with larger bites of 1 cm every 1 cm showed a reduction in hernias from 21% to 13% [15]. This reduction in the hernia rate may be due to an optimal distribution of tension across the wound [23,24]. A recent study in patients with laparotomic surgery for OC reported a reduction in the rate of incisional hernias of 17.2% to 7.9% using the small-bites technique [20,29]. Unfortunately, we did not systematically report the use of small-bites technique at the time of this study, and we cannot draw conclusions on this subject. Finally, some studies have described a major incidence of incisional hernias when using non-resorbable sutures for the fascia due to foreign body reaction. Consequently, there is an increased long-term infection risk as well as major local inflammation. Our results are consistent with the literature, despite statistical significance not being achieved.

In our study, negative pressure wound therapy was applied less frequently in the group of patients who developed a hernia. Negative pressure wound therapy in surgical wounds can improve healing and the restoration of dermal integrity, likewise minimizing the appearance of wound infections [30]. Chemotherapy has also been identified as an independent risk factor for hernias [20]. In this study, all patients underwent chemotherapy after surgery, and therefore, we could not analyze whether this treatment could have had an impact on the development of a hernia. Moreover, we do not have data on the timing of the initiation of the first postoperative cycle of chemotherapy, including the interval between surgery and the first cycle, as this information was not systematically recorded in our study cohort. Therefore, we are unable to determine whether the interval between surgery and the initiation of chemotherapy may have any relationship with the development of hernias.

In our multivariate analysis, the only risk factor independently associated with hernias was smoking. The mechanism for smoking to lead to hernias is multiple. Smoking reduces the blood flow and oxygen to tissues, which can weaken the abdominal wall and increase the risk of hernias. Nicotine can disrupt collagen synthesis, essential to tissue repair. Furthermore, smoking increases coughing, which raises intra-abdominal pressure. This combination of impaired healing and increased mechanical stress can make smokers more susceptible to developing hernias. Therefore, the preoperative detection of current smokers among patients provides an opportunity for smoking cessation or reduction and targeted respiratory prehabilitation, which can improve lung function and enhance oxygen delivery to tissues. By strengthening respiratory muscles, prehabilitation can decrease the incidence of persistent coughing and lower the likelihood of other respiratory issues, which could contribute to a lower incidence of hernias following surgery.

Adherence to an ERAS program was the only protective factor against the development of hernias in the multivariate analysis. This finding has never been demonstrated before. ERAS programs allow for the physical, nutritional, and psychological optimization of patients and have been shown to lead to early patient recovery, shorter hospital stays, and lower rates of readmission [21,31]. ERAS programs may contribute to lowering hernia rates by implementing a multifaceted approach to postoperative care. These programs promote early mobilization, which helps maintain muscle tone and reduces intra-abdominal pressure that can strain the abdominal wall. Optimized pain management minimizes the need for opioid use, which can reduce constipation and associated abdominal stress. ERAS protocols also focus on improving nutrition, ensuring patients receive adequate protein and the nutrients essential to wound healing. Additionally, ERAS minimizes surgical stress and enhances respiratory function through prehabilitation, which supports stronger tissue repair. This comprehensive approach not only accelerates recovery but could also fortify the abdominal wall, thereby significantly reducing the likelihood of hernia development post-surgery. However, in our study, the cohort of patients undergoing an ERAS program had been operated on later in time, and some of those patients had probably had the small-bites technique performed. This could be a potential bias to consider, but, unfortunately, this possibility could not be assessed in our study.

In our study, patients who followed the ERAS protocol underwent surgery starting in 2018 and therefore have a shorter follow-up period compared to patients who did not follow the ERAS protocol. Thus, the long-term impact of ERAS on reducing complications such as incisional hernias could become clearer with extended follow-ups. It is possible that some benefits or risks associated with ERAS might only become apparent over longer periods. For example, the improved tissue healing and reduced complication rates often associated with ERAS might translate into sustained protection against IH formation. Conversely, subtle differences in patient management or long-term tissue remodeling might lead to delayed complications not evident within a short follow-up.

The strengths of our study include the large number of patients, for which the known risk factors for incisional hernias were collected, and the availability of detailed information about the surgical closure technique of the abdominal wall in a population composed entirely of patients with OC and a laparotomic cytoreduction. Moreover, this is the first study that demonstrates that following an ERAS protocol can reduce the rate of post-operative hernias in these patients. This is an important finding that can enhance the routine performance of an ERAS program, especially in patients at higher risk of hernia development.

However, its limitations include the low number of events, which may affect the power to detect differences and the statistical validity of its associations. We did not perform a power analysis due to the retrospective nature of our study. The retrospective design introduces both information and selection bias. Moreover, the use of a small-bites technique was not registered, and therefore, it was not possible to establish the benefits of applying this technique in ovarian cancer. Additionally, the use of therapies such as Bevacizumab or PARP inhibitors was not recorded. This information is of great interest, as these drugs are currently part of the standard of care for ovarian cancer treatment, and their potential association with the development of hernias is particularly relevant and noteworthy. Last, the number of symptomatic patients with hernias is probably underestimated.

## 5. Conclusions

In conclusion, women that undergo cytoreductive surgery for advanced ovarian cancer are at an increased risk of having incisional hernias. The ERAS recovery program seems to be a protective factor against the development of hernias and should be routinely implemented in daily clinical practice. The preoperative identification of current smokers among the patients provides an opportunity for smoking cessation and targeted respiratory prehabilitation, which further contributes to the reduction in hernia rates. Since the incisional hernia is a common complication of cytoreductive surgery, with high morbidity and significant economic impact, randomized clinical trials should be performed to evaluate the prophylactic use of mesh or negative pressure wound therapy, among others, in selected cases.

## Figures and Tables

**Table 1:** Preoperative factors of patients with ovarian cancer undergoing cytoreductive surgery.

		Overall	No Hernia	Hernia	*p*-Value *	Missing (%)
n		156	126	30		
Age (mean (SD))		59.1 (12.4)	59.2 (12.9)	58.7 (10.4)	0.864	0.0
Type of surgery (%)	PDS	91 (58.3)	76 (60.3)	15 (50.0)	0.410	0.0
	IDS	65 (41.7)	50 (39.7)	15 (50.0)		
Smoker (%)	no	143 (91.7)	120 (95.2)	23 (76.7)	0.003	0.0
	yes	13 (8.3)	6 (4.8)	7 (23.3)		
BMI (median [IQR])		24.2 [21.95, 27.30]	23.9 [21.50, 27.30]	25.3 [23.42, 26.85]	0.133	0.0
Definitive histology (%)	clear-cell carcinoma	12 (7.7)	11 (8.7)	1 (3.3)	0.343	0.0
	dedifferentiated carcinoma	1 (0.6)	1 (0.8)	0 (0.0)		
	high-grade serous carcinoma	120 (76.9)	96 (76.2)	24 (80.0)		
	low-grade serous carcinoma	6 (3.8)	6 (4.8)	0 (0.0)		
	carcinosarcoma	1 (0.6)	1 (0.8)	0 (0.0)		
	endometrioid	11 (7.1)	7 (5.6)	4 (13.3)		
	squamous	1 (0.6)	1 (0.8)	0 (0.0)		
	mucinous	1 (0.6)	1 (0.8)	0 (0.0)		
	serous borderline	2 (1.3)	2 (1.6)	0 (0.0)		
	yolk sac	1 (0.6)	0 (0.0)	1 (3.3)		
DM (%)	no	147 (94.2)	117 (92.9)	30 (100.0)	0.284	0.0
	yes	9 (5.8)	9 (7.1)	0 (0.0)		
COPD (%)	no	149 (95.5)	121 (96.0)	28 (93.3)	0.880	0.0
	yes	7 (4.5)	5 (4.0)	2 (6.7)		
Corticosteroids treatment (%)	no	154 (98.7)	125 (99.2)	29 (96.7)	0.835	0.0
	yes	2 (1.3)	1 (0.8)	1 (3.3)		
IS (%)	no	154 (98.7)	124 (98.4)	30 (100.0)	1.000	0.0
	yes	2 (1.3)	2 (1.6)	0 (0.0)		
MDL (%)	LMA	22 (14.1)	17 (13.5)	5 (16.7)	0.269	0.0
	no	124 (79.5)	99 (78.6)	25 (83.3)		
	Pfannestiel	10 (6.4)	10 (7.9)	0 (0.0)		
Alb preqx (median [IQR])		4.10 [3.80, 4.30]	4.10 [3.80, 4.30]	4.10 [3.90, 4.20]	0.747	7.1
Prot preqx (median [IQR])		7.00 [6.60, 7.30]	7.00 [6.60, 7.40]	6.95 [6.60, 7.23]	0.791	5.8
Hb preqx (median [IQR])		11.90 [11.00, 13.20]	12.00 [11.05, 13.25]	11.65 [10.83, 13.00]	0.559	1.9
Ca 125 (median [IQR])		123.05 [31.10, 369.28]	128.10 [42.20, 394.20]	53.10 [25.00, 242.10]	0.210	1.3
Ascitis		59 (38.3)	52 (41.9)	7 (23.3)		
Vasc (median [IQR])		600.00 [200.00, 2350.00]	700.00 [200.00, 3000.00]	200.00 [200.00, 950.00]	0.279	1.3

* Comparison between groups using Wilcoxon and Chi-square test according to variable. DM: diabetes mellitus, COPD: chronic obstructive pulmonary disease. IS: immunosuppression; MDL: middle line laparotomy; Alb preqx: preoperative albumin; Prot preqx: preoperative proteins; Hb preqx: preoperative hemoglobin; Vasc: volume of ascites; IQR: interquartile range; SD: standard deviation.

**Table 2:** Intraoperative factors of patients with ovarian cancer undergoing cytoreductive surgery.

		Overall	No Hernia	Hernia	*p*-Value *	Missing
n		156	126	30		
Definitive FIGO Stage (%)	IIA	3 (1.9)	2 (1.6)	1 (3.3)	0.653	0.0
	IIB	9 (5.8)	6 (4.8)	3 (10.0)		
	IIIA	13 (8.3)	12 (9.5)	1 (3.3)		
	IIIB	14 (9.0)	10 (7.9)	4 (13.3)		
	IIIC	63 (40.4)	51 (40.5)	12 (40.0)		
	IVA	11 (7.1)	10 (7.9)	1 (3.3)		
	IVB	43 (27.6)	35 (27.8)	8 (26.7)		
Bowel resection (%)	0	55 (35.5)	46 (36.8)	9 (30.0)	0.415	0.6
	1	65 (41.9)	52 (41.6)	13 (43.3)		
	2	28 (18.1)	23 (18.4)	5 (16.7)		
	3	7 (4.5)	4 (3.2)	3 (10.0)		
Type of bowel resection (%)	anastomosis	96 (93.2)	76 (93.8)	20 (90.9)	0.996	34.0
	ostomy	7 (6.8)	5 (6.2)	2 (9.1)		
Blood loss (median [IQR])		400.00 [250.00, 750.00]	400.00 [237.50, 712.50]	425.00 [287.50, 762.50]	0.750	10.3
Surgical time (min) (median [IQR])		300.00 [240.00, 352.50]	300.00 [240.00, 342.50]	300.00 [255.00, 360.00]	0.419	7.7
Type of fascia suture (%)	monofilament	122 (98.4)	97 (98.0)	25 (100.0)	1000	20.5
	multifilament	2 (1.6)	2 (2.0)	0 (0.0)		
Type of fascia suture (%)	no resorbable	23 (18.7)	15 (15.0)	8 (34.8)	0.058	21.2
	absorbable	100 (81.3)	85 (85.0)	15 (65.2)		
Size of suture: number of 0 (mean (SD))	0	3 (3.0)	3 (3.7)	0 (0.0)	0.669	35.3
	1	39 (38.6)	32 (39.0)	7 (36.8)		
	2	59 (58.4)	47 (57.3)	12 (63.2)		
Fascia technique suturing (%)	continuous	59 (92.2)	51 (92.7)	8 (88.9)	1000	59.9
	single	5 (7.8)	4 (7.3)	1 (11.1)		
Type of cutaneous suture (%)	staples	54 (45.0)	39 (40.2)	15 (65.2)	0.053	23.1
	intradermic	66 (55.0)	58 (59.8)	8 (34.8)		
Subcutaneous drainage (%)	no	149 (96.1)	120 (96.0)	29 (96.7)	1000	0.6
	yes	6 (3.9)	5 (4.0)	1 (3.3)		
Abdominal drainage (%)	no	83 (53.5)	67 (53.6)	16 (53.3)	1000	0.6
	yes	72 (46.5)	58 (46.4)	14 (46.7)		
Negative pressure therapy (%)	no	75 (48.4)	55 (44.0)	20 (66.7)	0.043	0.6
	yes	80 (51.6)	70 (56.0)	10 (33.3)		

* Comparison between groups using Wilcoxon and Chi-square test according to variable. IQR: interquartile range; SD: standard deviation.

**Table 3:** Postoperative factors of patients with ovarian cancer undergoing cytoreductive surgery.

		Overall	No Hernia	Hernia	*p*-Value *	Missing
n		156	126	30		
Days of hospitalization (median [IQR])		6.00 [4.00, 9.00]	6.00 [4.00, 9.00]	7.00 [6.00, 10.00]	0.358	2.6
ERAS (%)	no	66 (45.5)	47 (39.8)	19 (70.4)	0.008	7.1
	yes	79 (54.5)	71 (60.2)	8 (29.6)		
Surgical infection (<30 d) (%)	no	122 (79.7)	101 (81.5)	21 (72.4)	0.405	1.9
	yes	31 (20.3)	23 (18.5)	8 (27.6)		
Infection localization (%)	abdominal abscess	11 (35.5)	10 (43.5)	1 (12.5)	0.144	80.1
	lymphocele	2 (6.5)	2 (8.7)	0 (0.0)		
	ascites	2 (6.5)	2 (8.7)	0 (0.0)		
	peritonitis	9 (29.0)	6 (26.1)	3 (37.5)		
	wound (skin and subcutaneous tissue)	7 (22.6)	3 (13.0)	4 (50.0)		
Wound dehiscence (%)	no	120 (78.9)	102 (82.9)	18 (62.1)	0.026	2.6
	yes	32 (21.1)	21 (17.1)	11 (37.9)		
Postoperative noradrenaline (%)	no	119 (76.8)	98 (78.4)	21 (70.0)	0.461	0.6
	yes	36 (23.2)	27 (21.6)	9 (30.0)		
ICU admission	no	146 (94.2)	119 (95.2)	27 (90.0)	0.510	
	yes	9 (5.8)	6 (4.8)	3 (10.0)		
Days in ICU (median [IQR])		10.00 [3.00, 21.00]	10.50 [4.75, 25.25]	3.00 [2.50, 12.00]	0.291	0.6
Blood transfusion (%)	no	67 (43.2)	56 (44.8)	11 (36.7)	0.547	0.6
	yes	88 (56.8)	69 (55.2)	19 (63.3)		
Nº concentrates (%)	0	67 (43.2)	56 (44.8)	11 (36.7)	0.349	0.6
	1	29 (18.7)	20 (16.0)	9 (30.0)		
	2	36 (23.2)	30 (24.0)	6 (20.0)		
	3	10 (6.5)	9 (7.2)	1 (3.3)		
	4	6 (3.9)	5 (4.0)	1 (3.3)		
	5	2 (1.3)	2 (1.6)	0 (0.0)		
	6	1 (0.6)	0 (0.0)	1 (3.3)		
	7	1 (0.6)	1 (0.8)	0 (0.0)		
	8	3 (1.9)	2 (1.6)	1 (3.3)		

* Comparison between groups using Wilcoxon and Chi-square test according to variable. ERAS: enhanced recovery after surgery, IQR: interquartile range; ICU: intensive care unit.

## Data Availability

The data presented in this study are available on request from the corresponding author.
